# The Impact of Social Loafing on Turnover Intention for Tourism Employees Post COVID-19: The Mediating Role of Mental Health

**DOI:** 10.3390/ijerph20095702

**Published:** 2023-05-01

**Authors:** Ibrahim A. Elshaer, Mohamed Algezawy, Mohanad M. S. Ghaleb, Shaimaa A. Mohamed, Alaa M. S. Azazz

**Affiliations:** 1 Management Department, College of Business Administration, King Faisal University, Al-Ahsaa 31982, Saudi Arabia; 2 Hotel Studies Department, Faculty of Tourism and Hotels, Suez Canal University, Ismailia 41522, Egypt; 3 Social Studies Department, College of Arts, King Faisal University, Al-Ahsaa 31982, Saudi Arabia; 4 Tourism Studies Department, Faculty of Tourism and Hotels, Mansoura University, Mansoura 35516, Egypt; 5 Tourism Studies Department, Faculty of Tourism and Hotels, Suez Canal University, Ismailia 41522, Egypt

**Keywords:** mental health, social loafing, turnover intention, tourism industry, PLS-SEM

## Abstract

The COVID-19 pandemic has led to widespread changes in the way that people work, including an increase in remote work and changes in group dynamics. Social loafing, the phenomenon of reduced individual effort in group settings, has been widely studied in the literature. However, less is known about the potential impacts of social loafing on mental health and turnover intention in this relationship. In this study, we hypothesized that social loafing would be related to turnover intention and that mental health would mediate this relationship. To test these hypotheses, we conducted a cross-sectional survey of 700 full-time tourism employees in Egypt. The obtained data were analyzed by Partial least squares structural equation modeling (PLS-SEM). Our results indicated that social loafing was significantly related to turnover intention and negative mental health consequences significantly mediated this relationship. The results showed that stress (as a dimension of mental health) experienced by employees may act as a mediator between social loafing and turnover intention. On the other hand, depression and anxiety were not observed to have a similar mediating effect. This implies that stress could play a vital role in the decision-making process of employees who are contemplating leaving their job due to social loafing. These findings suggest that interventions aimed at reducing social loafing may have the added benefit of improving mental health and decreasing turnover intention in the workplace.

## 1. Introduction

The COVID-19 pandemic has significantly altered how people work, including increased remote work and shifts in group dynamics [1]. These changes could potentially affect employee mental health and turnover intention. Social loafing is the phenomenon in which individuals put in less effort when working in a group compared to when working alone [2]. While social loafing can have negative impacts on productivity and the customer experience, it may also have negative impacts on the mental health of tourism industry employees. Research has found that social loafing can be a source of stress for employees, leading to reduced job satisfaction and increased turnover intention [3]. Additionally, social loafing is more likely to arise in work environments that have higher levels of psychological challenges and lower levels of social support [4]. This suggests that addressing social loafing in the tourism industry may not only improve productivity and customer satisfaction but may also have positive impacts on the well-being and mental health of employees.

Mental health is a critical aspect of overall well-being, and poor mental health can lead to negative outcomes; for example, reduced job satisfaction and boosted absenteeism [5]. Mental health is an increasingly significant concern in the tourism industry, as the industry is known for its high levels of stress and work-related demands [6]. Tourism industry employees may face unique challenges, such as irregular work schedules, long working hours, and high levels of customer interaction, all of which can contribute to mental health issues [7]. Turnover intention, or the intention to leave one’s current job, has also been related to negative consequences such as decreased organizational performance and increased costs [7,8,9]. Furthermore, turnover intention might be a consequence of poor mental health [6,7,9,10,11,12].

However, the relationship between social loafing, mental health, and turnover intention during the COVID-19 pandemic has not yet been extensively examined. It is important to investigate the relationship between social loafing, mental health, and turnover intention in the post COVID-19 pandemic workplace, as social loafing has the potential to impact not only individual performance but also the overall functioning and productivity of the group. Previous research has shown that social loafing can lead to negative outcomes such as decreased group cohesiveness and performance [13,14,15]. In addition, the negative effects of social loafing on mental health can have serious consequences, including increased absenteeism and decreased job satisfaction [3]. Understanding the mediating role of mental health in this relationship can also provide insight into how to mitigate the negative impacts of social loafing on turnover intention.

Additionally, the post-COVID-19-pandemic workplace may represent unique challenges and opportunities for addressing social loafing and its impact on mental health and turnover intention. The use of technology and virtual communication tools may provide new opportunities for promoting individual accountability and preventing social loafing [16]. Due to the challenges of monitoring and holding remote workers accountable for their work, social loafing may become more prevalent in remote work settings. On the other hand, the absence of physical presence in an office or workspace may reduce social pressure on remote employees to perform, leading to increased likelihood of procrastination or reduced effort [17]. The shift to remote work may affect group dynamics and the likelihood of social loafing occurring, as the lack of physical proximity can make it more difficult for group members to monitor and evaluate each other’s contributions [1,7,18,19]. This contradictory argument presents an interesting research question: has social loafing increased or decreased post COVID-19? The answer to this question could have significant implications for organizations seeking to maintain remote work arrangements in the long term. This unique context presents an opportunity to test the impact of social loafing on mental health.

The self-determination theory (SDT) can help in understanding the influence of social loafing on tourism employees’ mental health. According to SDT, individuals have innate psychological wants for competence, autonomy, and relatedness [20]. When these desires are met, individuals experience greater well-being and motivation. Social loafing in tourism teams can undermine the fulfillment of these needs. For example, social loafing can lead to a lack of autonomy as individuals feel less control over their work and a lack of competence as individuals feel their contributions are undervalued [21,22,23,24]. Additionally, social loafing can lead to a lack of relatedness as individuals feel disconnected from their team and less likely to form positive social connections [24,25]. Furthermore, studies have shown that when these needs are not met, individuals may experience negative effects on their mental health, such as burnout and reduced job satisfaction [8,26]. Thus, SDT suggests that social loafing can have a detrimental impact on the mental health of tourism employees by undermining the fulfillment of their psychological needs.

The current research paper aims to contribute to the existing literature by examining for the first time the meditating role of employees’ mental health in the relationship between social loafing and turnover intention post COVID-19 pandemic, a unique and unprecedented situation that has caused a widespread change in the way that people work. The COVID-19 pandemic has brought unprecedented challenges to the tourism industry, with many companies experiencing significant financial losses and a reduced workforce. In such an environment, it is critical for organizations to understand the factors that impact employee turnover intention, particularly social loafing and mental health. By understanding this relationship, we can identify potential interventions and strategies for promoting mental health and reducing turnover intention in the post-pandemic workplace.

The next part of the research further develops the conceptual framework regarding social loafing, mental health, and turnover intention. The research material, methods, and data analysis techniques are described in Section 3, and the results are presented in Section 4. Finally, the discussion and their implications are elaborated in Section 5, while the conclusion section and the limitations and further opportunities for research are outlined in Section 6.

## 2. Theoretical Background

### 2.1. Social Loafing and Turnover Intention

Turnover intention is the intention of an employee to leave their current job [25]. The hospitality industry often experiences high turnover due to negative perceptions of the industry and low compensation [27]. Studies have identified several factors that can predict turnover intention in the hospitality industry, such as organizational support [28], organizational citizenship behavior [29], organizational justice [30,31,32], job satisfaction and organizational commitment [5], coworkers and job security [9,33,34,35], and leadership style and social loafing [9,36]. The social theory of social loafing suggests that people are more likely participate in social loafing when they believe that their efforts will not be noticed or that their contributions will not make a significant impact. The theory was first introduced by Max Ringelmann in 1913 as cited in [37], who conducted a series of experiments involving rope-pulling tasks. Ringelmann found that the more individuals he added to a rope-pulling task, the less effort each person exerted, resulting in a decrease in overall performance. Subsequent research has confirmed the existence of social loafing and has identified a number of factors that can contribute to its occurrence. For example, social loafing is more likely to occur when individuals believe that their contributions will not be noticed or evaluated [38]. Additionally, social loafing frequently arises in large groups or when individuals feel anonymous within the group [39]. The social theory suggests that group members influence each other’s behaviors and that a group member who perceives that other team members are not putting in enough effort may reduce their own effort as well [40]. This can foster the phenomenon called social loafing, in which an individual’s motivation decreases and they put in reduced effort when working in a group setting [25,41]. Social loafing has been linked to low-performing workers and turnover intention [42]. Recent research has found that social loafing behavior positively influences turnover intention among restaurant employees [9]. Based on this, it can be hypothesized that:

**H1:**  *Social loafing significantly impact turnover intention.*

### 2.2. Social Loafing and Mental Health (Depression, Anxiety, and Stress)

The tourism industry is known for its higher levels of stress and work-related challenges [7,35,43], which can contribute to mental health issues including anxiety, depression, and stress between employees. The COVID-19 pandemic has created significant insecurity and stress for employees in the tourism industry [44], as well as for businesses. This uncertainty can lead to decreased employee motivation and engagement [45], making it important to understand and address the possible impact of social loafing on turnover intention. Furthermore, the COVID-19 pandemic has led to a significant increase in remote work and virtual collaboration [46], which can increase the potential for social loafing. With employees working from home, it can be more difficult to monitor and manage individual performance, making it important to understand the impact of social loafing on employee motivation and engagement.

Tourism industry employees may face unique challenges such as irregular work schedules, long working hours, and high levels of customer interaction, all of which can contribute to mental health issues [47,48]. Studies have found that tourism industry employees are at an increased risk of developing anxiety, depression, and stress compared to employees in other industries [49,50,51]. Social loafing can greatly impact the well-being and mental health of tourism industry employees. Research has found that social loafing can be a source of stress for employees, leading to reduced job satisfaction [52]. Additionally, social loafing repeatedly arises in work environments that have higher levels of psychological pressures and lower levels of social support [4]. When individuals believe that their exerted endeavors are not being recognized or valued, they may be less motivated to put in effort, which can lead to feelings of depression, stress, and anxiety [4,50]. Additionally, social loafing may lead to an overall decrease in productivity and a negative impact on the customer experience, which can further contribute to stress and mental health issues among employees [22]. Furthermore, workplace anxiety and depression are common mental health issues that can have significant impacts on the well-being of tourism industry employees and on the performance and success of tourism businesses [9]. Social loafing may be a contributing factor to these issues. Accordingly, we ca propose the below hypotheses:

**H2:**  *Social loafing significantly impact employee stress.*

**H3:**  *Social loafing significantly impact employee anxiety.*

**H4:**  *Social loafing significantly impact employee depression.*

### 2.3. Mental Health (Depression, Anxiety, and Stress) and Turnover Intention

The COVID-19 pandemic has had a significant effect on the tourism industry, leading to widespread job loss and economic uncertainty for many employees [3,27,53]. The COVID-19 pandemic has also disrupted the industry in several ways, including travel restrictions, reduced demand, and changes in consumer behavior [47]. These changes may increase the workload and stress levels of tourism employees, leading to an increase in turnover intention and mental health issues [35]. These stressors may contribute to an increase in workplace anxiety and depression among tourism employees. The hospitality industry frequently acknowledges the detrimental effects of voluntary turnover intentions [45,54]. Previous studies have shown that tourism employees who experience depression have a stronger desire to leave their job [55,56]. Research also supports the idea that employees in high-stress positions, such as hotel workers, are more likely to have increased turnover intention [57,58]. As stated by the authors of [59], the perception of job stress is negatively associated with job satisfaction and positively related to the intention of turnover. Fletcher [60] discovered that excessive stress at work can lead to physical and psychological difficulties and have a negative impact on the intention of turnover. Cummins [61] found that a high perception of occupational stress is significantly related to poor mental health, including employee dissatisfaction with their job. Mikkelsen et al. [62] reported that occupational stress can lead to organizational dysfunctions, such as lower levels of organizational commitment, higher level of absenteeism, and higher intention of turnover. Barsky et al. [63] also found that occupational stress is a predictor of various negative attitudes and behaviors, such as a higher level of turnover intention and lower levels of job satisfaction. Moreover, Karatepe et al. [3] discovered that a high level of turnover intention can be caused by occupational stress and recommended enhancing work conditions to enhance job performance and reduce the likelihood of employees leaving their job. Additionally, the global panic caused by the COVID-19 pandemic and the implementation of strict safety measures brought about anxiety and frustration [35,38,58], particularly among workers in the service sector (including hotels and travel agents). As a result, it is predicted that increased anxiety in the workplace during the pandemic will lead to decreased job satisfaction and job motivation among hospitality workers and heightened turnover intentions. Consequently, it can be proposed that mental health—specifically, depression, anxiety, and stress—has a positive correlation with turnover intention as below:

**H5:**  *Stress significantly impacts employee turnover intention.*

**H6:**  *Anxiety significantly impacts employee turnover intention.*

**H7:**  *Depression significantly impacts employee turnover intention.*

### 2.4. The Mediating Role of Employees’ Mental Health in the Relationship between Social Loafing and Turnover Intention

In the tourism industry, social loafing can have negative results for both the employees and the organization. It may lead to a decline in customer satisfaction and an increase in the workload for other employees. Social loafing can increase the employee’s turnover intention [9,36,42]. However, the link between social loafing and the intention of turnover is not straightforward and may be influenced by other factors, such as employees’ mental health (stress, depression, and anxiety). Mental health issues, such as stress, depression, and anxiety, can impact an individual’s job satisfaction, engagement, and performance, and we propose that it can mediate the relationship between social loafing and turnover intention. For example, employees who experience high levels of social loafing in their work environment may also experience increased stress, depression, anxiety [4,50,52] and decreased job satisfaction, leading to increased turnover intention. On the other hand, employees who have positive mental health and are less susceptible to the negative effects of stress and depression may be less likely to be impacted by social loafing and may have lower levels of turnover intention.

**Figure 1:**
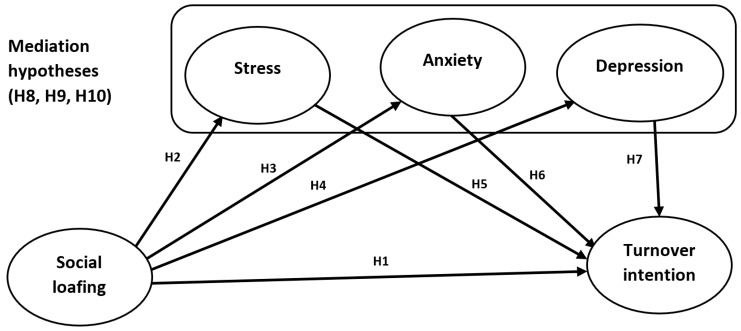
A research framework.

Moreover, when employees experience higher levels of stress, depression, or anxiety, the negative effects of social loafing on turnover intention may be amplified. For example, if an employee is already feeling stressed or anxious, they may be more likely to become disengaged and reduce their effort when working in a group. This can lead to a decrease in productivity and job satisfaction, which can ultimately contribute to a higher intention to leave. Therefore, it is essential for organizations to consider the potential mediation impact of mental health on the relationship between social loafing and turnover intention. By prioritizing mental health and providing resources to support employees’ well-being, companies can reduce the negative effects of social loafing and ultimately improve employee retention. Hence, we can propose the below hypotheses (as seen in Figure 1):

**H8:**  *Stress mediates the impact of social loafing on turnover intention.*

**H9:**  *Anxiety mediates the impact of social loafing on turnover intention.*

**H10:**  *Depression mediates the impact of social loafing on turnover intention.*

## 3. Materials and Methods

### 3.1. Sampling and Data Collection

In November and December of 2022, a survey was given to 730 full-time workers employed in five-star hotels and category-A travel agents in two main cities in Egypt (Cairo, the capital, and the well-known resort city Sharm El Sheikh). In Egypt, travel agents are classified into three categories based on their business size, performance, and level of services. Category-A travel agents are considered the largest and most established companies in the travel industry in Egypt. These travel agents offer a wide range of services to their clients, including flight bookings, hotel reservations, tour packages, and transportation services. They have extensive networks and partnerships with airlines, hotels, and tour operators worldwide, allowing them to provide comprehensive travel solutions to their customers. The employees in the tourism industry were targeted because this field often requires teamwork in large cross-functional groups, which can raise the likelihood of social loafing due to the perception that one’s individual efforts will not make a significant impact. Furthermore, the tourism industry is seasonal and experiences changes in demand and employee workload, making it difficult for companies to maintain consistent levels of employee motivation and engagement and, therefore, increasing the possibility of social loafing.

The researchers designed (Google forms) and distributed the survey link using their personal networks via social media platforms and were able to collect a high response rate of 95% by using this method [64]. Of the 730 questionnaires distributed, 708 were returned and 8 were discarded due to incomplete answers, resulting in 700 usable questionnaires for analysis. The sample size of 700 is considered sufficient for PLS-SEM tests as it meets the requirements for adequate sample size in this type of study. One of the core concerns in PLS-SEM is determining the minimum sample size. The “10-times rule” method, popularly used for estimating minimum sample size in PLS-SEM as suggested by Hair et al. [65], is based on the premise that the sample size used in an empirical study should be at least 10 times larger than the highest number of inner or outer model links associated with any latent variable in the model. As we have 7 latent dimensions with 28 reflective variables (total of 35), the minimum sample size is 350, and our sample size of 700 is more than enough. A t-test was run to check whether there were any notable variations in the mean answers between participants who filled out the survey early and those who filled it out later. The results revealed that there were no significant differences between the means, implying that non-response bias might exist in the study as stated by Bryman and Cramer [66].

### 3.2. Study Measures

TThe scales utilized in this paper were selected based on their established psychometric properties and derived from previous literature. The survey was designed with a multi-item scale that used 5 Likert scales to measure the constructs of the study. Employees mental health was evaluated using the Depression, Anxiety and Stress Scale-21 Items (DASS-21) scale, which measures depression, anxiety, and stress with 21 items and is easy to use in both clinical and non-clinical research. The DASS-21 is often used to identify negative emotions experienced by individuals [67]. Each dimension of the DASS-21 has 7 sub-items. Similarly, the researchers used 4 questions to measure social loafing. The items were adapted from Price, Harrison, and Gavin [68]. Participants were asked to rate the likelihood that each of their coworkers exhibited these behaviors on a 5-point scale, where 1 means highly likely to loaf and 5 means highly unlikely to loaf. The turnover intention was assessed using 3 reflective items developed by the authors of [3,32] which reflect a desire to leave one’s current job and find a new one.

The reason for employing a 5-point scale in our study is because it allows respondents to make clearer decisions when given fewer options, which can lower the chance of confusion and reduce the likelihood of errors in their responses. Additionally, Dawes [69] conducted a study where he compared three different types of scales, with the 5- and 7-point formats being the most widely used [70]; the 10- or 11-point scales are also commonly employed [71]. He found that none of the scales generated data with significantly lower variance around the mean, indicating that none of the three formats are inferior in terms of obtaining data that can be utilized for regression analysis.

To reduce the risk of common method variance (CMV) in our self-report study, several ex ante techniques were utilized in the research design as suggested by Lindell and Whitney [72], Witteloostuijn et al. [73], and Podsakoff et al. [74]. Procedures were put in place during the questionnaire development process to minimize this bias [74]. For instance, the dependent variables were positioned before the independent variables as suggested by [74] and the participants’ anonymity and confidentiality were ensured. The questionnaire questions were piloted by nine academics and fifteen employees to ensure its reliability and clarity. No changes were made to the questionnaire after the pilot test. The survey was written in English and then professionally translated into Arabic (the participants’ native language) and back into English. Finally, a Harman’s single-factor analysis was conducted. The extracted factors were set to a value of 1.00 in an exploratory factor analysis test using Statistical Package for the Social Sciences (SPSS), New York, Unites States, with no rotation. Only one factor emerged, which explained 43% of the variance. This means CMV is not a problem in our study.

### 3.3. Data Analysis Methods

PLS-SEM was employed in order to examine the proposed theoretical postulations which include the role of mental health as a mediator in the association between social loafing and turnover intention among tourism employees. PLS-SEM is a Variance-Based Structural Equation Modeling (VB-SEM) method. VB-SEM tests assumptions that are based on theory but are data-driven, while Covariance-Based Structural Equation Modeling (CB-SEM) is strictly theory-driven [75]. Our study is based on integrating (for the first time) two previous theories (social loafing theory and self-determination theory), making VB-SEM a better fit than CB-SEM in our study. Additionally, the CB-SEM method is a confirmatory method for an existing theory, while VB-SEM orientation is predictive in its nature [76]. The aim of the current study is to test the capability of social loafing in predicting turnover intention through the mediating role of mental health; thus, VB-SEM is a more suitable choice than CB-SEM.

The study used Leguina’s [77] two-step technique which includes evaluating the measurement model for reliability and validity and then assessing the structural model to either support or refute the hypotheses. Various recommended cut-off values were utilized to evaluate the outer model in PLS-SEM, such as a “standardized factor loading” (FL > 0.7), “composite reliability” (CR > 0.7), “average variance extracted” (AVE > 0.5), “normed fit index” (NFI > 0.9), standardized root mean square residual (SRMR < 0.8), R^2^ > 0.1, and Stone–Geisser Q^2^ > 0.0 as per the recommendations of Hair et al. [78].

## 4. Results

### 4.1. Descriptive Results

The research found that 65% of the study participants were male and 35% were female. The higher representation of men compared to women in our study sample may be due to the nature of the tourism industry, which is characterized by long and irregular working hours which can be particularly challenging for women who are also responsible for caregiving duties. When it comes to age, 20% of the participants were under 20, 41% were between 26 and 35, 31% were between 36 and 45, and 8% were 46 or older. These findings indicate that the majority of employees in the Egyptian tourism industry (61%) are young and capable of performing physically demanding work. In terms of education, 50% of the participants had completed secondary school or less, 45% had an undergraduate degree, and just 5% had a postgraduate degree. A total of 60% (420) of respondents were from five-star hotels, and 40% (280) were from category-A travel agents. For their roles, 61% worked in front-line positions and 39% worked in back-line positions. In regards to experience, 20% had 1 year or less of experience, 21% had 2–4 years, 35% had 5–7 years, and 24% had 8 years or more.

### 4.2. Measurements Outer Model Evaluation

Tests were performed to evaluate the validity and reliability of the inner model (measurement model) in the study, as indicated in Table 1. These tests included composite reliability (C.R.), internal consistency Cronbach’s alpha (α), and discriminant and convergent validity (for construct validity). Table 1 shows that the Cronbach’s alpha, composite reliability, and average variance extracted (AVE) scores for the latent unobserved dimensions of social loafing (α = 0.838, C.R. = 0.838, AVE = 0.674), turnover intention (α = 0.890, C.R. = 0.896, AVE = 0.820), stress (α = 0.971, C.R. = 0.974, AVE = 0.854), anxiety (α = 0.974, C.R. = 0.976, AVE = 0.866), and depression (α = 0.965, C.R. = 0.968, AVE = 0.829) all exceeded the recommended cutoff level. These results indicate that the study has good internal reliability and convergent validity of the constructs.

Furthermore, the standardized factor loadings (SFL) were all found to be above 0.70, providing additional support for the scale’s reliability as per Kline [79]. The study also evaluated the discriminant validity using three methods as suggested by Leguina [77]: cross-loading, Fornell–Larcker, and heterotrait–monotrait matrix. The findings, shown in Table 2, demonstrate that the items for each latent variable had loadings that were higher than the cross-loadings with other scale items, indicating that each latent variable is distinct from the other.

Besides cross-loading and Fornell–Larcker matrix evaluations, the study also examined the discriminant validity using the heterotrait–monotrait ratio test. The findings presented in Table 3 showed that the squared average variance extracted values for each latent variable are higher than the inter-variable correlation coefficients, indicating that the constructs are different from each other. In addition, the values for the heterotrait–monotrait ratio were less than 0.90, as suggested by Leguina [77]. These results demonstrate that the measures used in the study have good reliability, discriminant validity, and convergent validity, and thus the inner structural model can be analyzed for hypothesis assessment.

### 4.3. Inner Model Assessment (Hypotheses Testing)

After the measurement model was found to have good convergent and discriminant validity, the structural model was analyzed to examine its ability to predict and explain the impacts of the exogenous latent unobserved variables on the endogenous unobserved dependent latent variables [80]. A number of metrics were used to evaluate the goodness of fit (GoF) of the model. To ensure a good fit, the minimum acceptable R^2^ score should be 0.10 as per Hair et al. [78]. The results reveal that the endogenous latent variables of anxiety, depression, stress, and turnover intention have R^2^ values of 0.324, 0.355, 0.348, and 0.590, respectively, indicating that the model has good predictive power. Additionally, the Stone–Geisser Q^2^ criterion showed values of 0.384 for turnover intention, 0.346 for stress, 0.322 for anxiety, and 0.351 for depression, further supporting the model’s ability to make accurate predictions as per Henseler et al. [81]. Furthermore, the GoF can be calculated by taking the square root of the average of all R^2^ values multiplied by the average of all AVE values. The GoF testing resulted in a value of 0.707, indicating a large goodness of model fit as suggested by Wetzels et al. [82].

**Figure 2:**
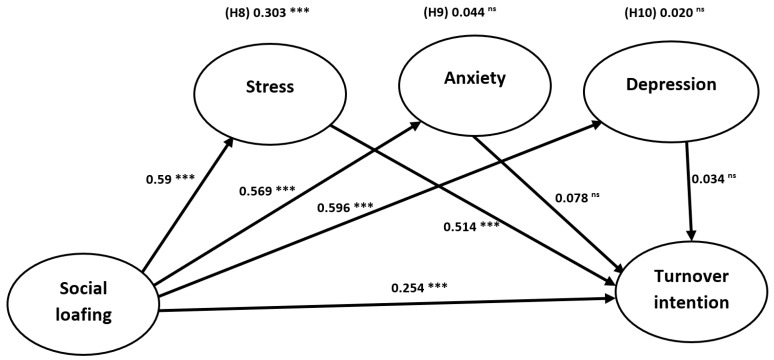
The model results. *** Significance level below 0.001; ns: non-significant.

In the final step of the analysis, a bootstrapping approach with 5000 iterations was used to evaluate the path coefficient effects and t-significance values for the direct and mediating relationships, as shown in Table 4 and Figure 2. The study proposed and tested seven direct and three mediating hypotheses. The results revealed that social loafing had a positive and significant direct effect on turnover intention (β = 0.254, t = 8.554, *p* < 0.001), stress (β = 0.590, t = 27.242, *p* < 0.001), anxiety (β = 0.569, t = 23.00, *p* < 0.001), and depression (β = 0.596, t= 23.816, *p* < 0.001) in support of hypotheses H1, H2, H3, and H4. Additionally, stress had a positive and significant direct effect on turnover intention (β = 0.514, t = 8.825, *p* < 0.001), which supports H5. Interestingly, anxiety (β = 0.078, t = 1.897, *p* > 0.05) and depression (β = 0.034, t = 1.067, *p* > 0.05) failed to significantly impact turnover intention. Therefore, H6 and H7 were not supported.

For the mediation effects, the PLS-SEM analysis revealed that stress played a mediating role in the link between social loafing and turnover intention (β = 0.303, t = 8.131, *p* < 0.001), supporting H8. However, anxiety (β = 0.044, t = 1.883, *p* > 0.05) and depression (β = 0.020, t = 1.070, *p* > 0.05) failed to mediate the link between social loafing and turnover intention; therefore, H9 and H10 were not supported.

## 5. Discussion

The tourism industry is one of the largest industries in the world and employs millions of people globally [83]. However, despite its size and importance, the industry is characterized by high levels of stress, depression, and anxiety among its employees, particularly among front-line employees [3,6] such as front-desk staff and travel agency workers. High levels of mental health issues can have serious consequences for both employees and their employers, including decreased job satisfaction, reduced productivity, and increased absenteeism. One factor that has been identified as contributing to high levels of stress, depression, and anxiety among tourism employees is social loafing. Social loafing indicates the propensity of individuals to reduce their effort when they are working in a group compared to when they are working alone [39]. This phenomenon is particularly relevant to the tourism industry, where employees often work in teams and are collectively responsible for the success of a tour or hospitality service.

Social loafing and turnover intention are two prevalent phenomena in the tourism industry that can have a significant impact on employee well-being. Studies have shown that employees who experience social loafing in their workplace are at an increased risk of stress, depression, and anxiety [49,84,85]. Similarly, turnover intention can be a result of poor mental health outcomes among tourism employees [6,12,34,86]. However, it is not clear how these two phenomena are related and how they might interact to impact employee turnover intention.

The findings of this paper imply that social loafing is a significant factor contributing to high turnover intentions among tourism employees. Social loafing can reduce employee morale and job satisfaction, leading to increased intentions to leave their current employment. This is particularly concerning for tourism organizations, as high turnover rates can result in increased costs, reduced productivity, and decreased employee morale and job satisfaction. The results of the PLS-SEM analysis showed that social loafing had a significant and positive impact on turnover intention, stress, anxiety, and depression as predicted by hypotheses H1, H2, H3, and H4. Additionally, the analysis showed that stress had a significant and positive effect on turnover intention, supporting hypothesis H5. However, anxiety and depression were found to have no significant impact on turnover intention, meaning hypotheses H6 and H7 were not supported.

An intriguing result that emerged from our study is that only employees’ stress (as a dimension of employee’s mental health) plays a mediating role in the connection between social loafing and turnover intention (H8), while depression and anxiety (mental health dimensions) did not show the same mediating effect (H9 and H10). This suggests that stress may play a significant role in the decision-making process of employees who are considering leaving their job due to social loafing. While stress partially mediates the relationship between social loafing and turnover intentions, depression and anxiety did not show the same effect. This implies that, although stress has a significant impact on the connection between social loafing and turnover intentions, there are additional factors beyond depression and anxiety—such as job satisfaction, work-life balance, and job security—that contribute to the relationship as well. Moreover, the tourism industry is recognized for its elevated levels of job-related depression and anxiety because of its unstable nature [35,43] and the ongoing health crisis [12,18,44]. These conditions can be intensified by the nature of the job, including long work hours, a fast-paced work environment, and the need to interact with numerous people. However, tourism employees may have a higher tolerance for depression and anxiety and be more resilient to its effects, which could help to explain the insignificant relationship between depression, anxiety, and turnover intentions.

## 6. Conclusions

The interconnection among social loafing, mental health, and turnover intention is intricate and diverse, particularly in the tourism sector, where high stress and workloads can adversely affect employee health. Social loafing can exert a considerable influence on turnover intention, and mental health is a crucial factor in studying the relationship between social loafing and turnover intention among tourism employees. Employers in the tourism industry should strive to create supportive and healthy work environments that promote employee well-being, which can help to reduce social loafing and turnover intention. The results of the current study showed that social loafing had a significant and positive impact on turnover intention. Additionally, the analysis showed that only the stress dimension of mental health had a significant and positive effect on turnover intention. However, anxiety and depression were found to have no significant impact on turnover intention. This suggests that, while stress has a significant impact on turnover intention, tourism employees may be more resilient to the effects of depression and anxiety, which could explain the insignificant relationship between these two factors and turnover intention. This study also investigated the mediating role of mental health in the relationship between social loafing and turnover intention for the first time. The PLS-SEM analysis revealed that stress played a mediating role in this relationship. However, anxiety and depression were not found to mediate the link between social loafing and turnover intention. This indicates that stress might have a considerable influence on the thought process of employees who are thinking of quitting their job because of social loafing.

### 6.1. Implications

Our study provided several theoretical and practical implications. Theoretically, the findings of the study suggest that social loafing and turnover intention are not independent constructs, but are related to each other, and that employee’s mental health (stress, depression, and anxiety) may play a mediating role in the relationship between social loafing and turnover intention. This highlights the need for more research on the interactions between different workplace phenomena and their impact on employee well-being. The study has several practical implications as well for organizations in the tourism industry. Firstly, it suggests that organizations should focus on promoting a culture of accountability and teamwork in order to mitigate social loafing. This can be done by encouraging employee participation and providing regular feedback [67]. Secondly, organizations should focus on reducing turnover intention by providing opportunities for professional development and career advancement. This can lead to an improvement in employee satisfaction and decrease the possibility of negative impacts on mental health. Furthermore, the study suggests that organizations should view turnover intention as an indicator of potential disengagement and lack of motivation among their employees, which could also be an early warning of negative mental health issues. It is important for organizations to be aware of this indicator and take proactive measures to prevent it from impacting their employees’ mental well-being.

### 6.2. Limitations and Future Research Opportunities

Similar to other studies, our study has some limitations. The generalizability of the findings in other cultural contexts, causality, and temporal ordering could be questioned. Additionally, while our cross-sectional studies have found a relationship between social loafing, mental health, and turnover intention, it is unclear how this relationship evolves and changes over time. Therefore, longitudinal studies are needed to fully understand the dynamic nature of these relationships. Future studies should employ different research designs, examine the generalizability of the findings across different cultural contexts, and include other factors (i.e., job satisfaction and commitment) that may mediate or moderate the impact of social loafing on employee turnover intention. Future studies might examine the effect of other interventions aimed at reducing social loafing and turnover intention and improving employee’s mental health in the tourism industry.

## Figures and Tables

**Table 1:** Calculations of the outer model.

Factors/Items	St. Loadings	α	C.R.	AVE
Thresholds Points	>0.7	>0.7	>0.7	>0.5
Anxiety		0.974	0.976	0.866
Anzi_1: I noticed my mouth was dry	0.947			
Anzi_2: I had trouble breathing (such as rapid or shortness of breath without exertion)	0.924			
Anzi_3: I felt shaking in my hands	0.921			
Anzi_4: I was concerned about potentially panicking and embarrassing myself	0.923			
Anzi_5: I felt close to a panic attack	0.947			
Anzi_6: I felt scared without a clear reason	0.938			
Anzi_7: I became aware of my heartbeat without any physical activity (such as feeling my heart rate increase or skipping a beat)	0.914			
Depression		0.965	0.968	0.829
Dprsn_1: I was unable to feel any happiness or positive emotions	0.956			
Dprsn_2: I struggled to start tasks and take initiative	0.891			
Dprsn_3: I saw no reason to be optimistic or have hope	0.891			
Dprsn_4: I felt sad and depressed	0.890			
Dprsn_5: I didn’t feel good about myself or my worth	0.934			
Dprsn_6: I was unable to get excited or passionate about anything	0.905			
Dprsn_7: I felt that life had no purpose or significance	0.904			
Social Loafing		0.838	0.838	0.674
Loaf_1: I let others do the work for me	0.745			
Loaf_2: I made excuses about having other issues to do when someone needed support	0.839			
Loaf_3: I shirked work and shirked my responsibilities	0.830			
Loaf_4: I didn’t do my fair share of the tasks	0.866			
Stress		0.971	0.974	0.854
Strs_1: I had trouble calming down and relaxing	0.972			
Strs_2: I tended to have extreme reactions to events	0.914			
Strs_3: I felt I was employing a lot of nervous energy	0.913			
Strs_4: I became easily irritated	0.916			
Strs_5: I had difficulty finding relaxation	0.951			
Strs_6: I became easily frustrated by anything that disrupted my plans	0.967			
Strs_7: I felt I was easily upset or quick to anger	0.829			
Turnover Intention		0.890	0.896	0.820
Turn_Intn_1: I frequently consider quitting my career	0.914			
Turn_Intn_2: It wouldn’t take much to push me to leave my current career	0.939			
Turn_Intn_3: I am likely to start searching for a different career soon	0.863			

**Table 2:** Cross-loadings.

	Anxiety	Depression	Social Loafing	Stress	Turnover Intention
Anzi_1	0.947	0.550	0.560	0.497	0.475
Anzi_2	0.924	0.528	0.497	0.432	0.431
Anzi_3	0.921	0.535	0.493	0.451	0.435
Anzi_4	0.923	0.480	0.516	0.450	0.472
Anzi_5	0.947	0.573	0.587	0.527	0.502
Anzi_6	0.938	0.554	0.541	0.484	0.474
Anzi_7	0.914	0.459	0.504	0.436	0.467
Dprsn_1	0.559	0.956	0.596	0.683	0.603
Dprsn_2	0.494	0.891	0.571	0.642	0.550
Dprsn_3	0.487	0.891	0.532	0.620	0.475
Dprsn_4	0.478	0.890	0.510	0.599	0.473
Dprsn_5	0.529	0.934	0.567	0.654	0.572
Dprsn_6	0.542	0.905	0.511	0.624	0.529
Dprsn_7	0.510	0.904	0.501	0.607	0.523
Loaf_1	0.529	0.523	0.745	0.481	0.493
Loaf_2	0.434	0.499	0.839	0.549	0.543
Loaf_3	0.447	0.423	0.830	0.422	0.469
Loaf_4	0.453	0.500	0.866	0.473	0.528
Strs_1	0.491	0.654	0.586	0.972	0.738
Strs_2	0.452	0.642	0.529	0.914	0.629
Strs_3	0.451	0.641	0.530	0.913	0.635
Strs_4	0.465	0.645	0.521	0.916	0.668
Strs_5	0.489	0.654	0.557	0.951	0.699
Strs_6	0.481	0.653	0.566	0.967	0.728
Strs_7	0.432	0.619	0.526	0.829	0.589
Turn_Intn_1	0.451	0.526	0.576	0.663	0.914
Turn_Intn_2	0.492	0.573	0.585	0.696	0.939
Turn_Intn_3	0.415	0.493	0.527	0.613	0.863

**Table 3:** Calculations of discriminant validity.

	AVE Scores					Heterotrait-Monotrait Ratio (HTMT) Scores				
	1	2	3	4	5	1	2	3	4	5
1. Anxiety	0.931									
2. Depression	0.566	0.910				0.581				
3. Social Loafing	0.569	0.596	0.821			0.627	0.658			
4. Stress	0.505	0.696	0.590	0.924		0.517	0.719	0.651		
5. Turnover Intention	0.501	0.587	0.622	0.727	0.906	0.536	0.630	0.717	0.779	

**Table 4:** Hypotheses evaluation.

Direct and Specific Indirect Effects	β	t-Value	*p*-Value	Results
Direct Effects				
H1: Social Loafing → Turnover Intention	0.254	8.554	0.000	Supported
H2: Social Loafing → Stress	0.590	27.242	0.000	Supported
H3: Social Loafing → Anxiety	0.569	23.00	0.000	Supported
H4: Social Loafing → Depression	0.596	23.816	0.000	Supported
H5: Stress → Turnover Intention	0.514	8.825	0.000	Supported
H6: Anxiety → Turnover Intention	0.078	1.897	0.058	Not Supported
H7: Depression → Turnover Intention	0.034	1.067	0.286	Not Supported
Specific Indirect Effects				
H8: Social Loafing → Stress → Turnover Intention	0.303	8.131	0.000	Supported
H9: Social Loafing → Anxiety → Turnover Intention	0.044	1.883	0.060	Not Supported
H10: Social Loafing → Depression → Turnover Intention	0.020	1.070	0.285	Not Supported

## Data Availability

Data is available upon request from researchers who meet the eligibility criteria. Kindly contact the first author privately through e-mail.
